# A novel Cas9-targeted long-read assay for simultaneous detection of *IDH1/2* mutations and clinically relevant *MGMT* methylation in fresh biopsies of diffuse glioma

**DOI:** 10.1186/s40478-020-00963-0

**Published:** 2020-06-20

**Authors:** Thidathip Wongsurawat, Piroon Jenjaroenpun, Annick De Loose, Duah Alkam, David W. Ussery, Intawat Nookaew, Yuet-Kin Leung, Shuk-Mei Ho, John D. Day, Analiz Rodriguez

**Affiliations:** 1 Department of Biomedical Informatics, College of Medicine, University of Arkansas for Medical Sciences, Little Rock, AR 72205 USA; 2 Department of Neurosurgery, College of Medicine, University of Arkansas for Medical Sciences, Little Rock, AR 72205 USA; 3 Department of Pharmacology and Toxicology, College of Medicine, University of Arkansas for Medical Sciences, Little Rock, AR 72205 USA

**Keywords:** Glioblastoma, *MGMT*, *IDH*, Targeted sequencing, CRISPR/Cas9, Long-read sequencing, Nanopore, Molecular marker, Methylation

## Abstract

Molecular biomarkers provide both diagnostic and prognostic results for patients with diffuse glioma, the most common primary brain tumor in adults. Here, we used a long-read nanopore-based sequencing technique to simultaneously assess *IDH* mutation status and *MGMT* methylation level in 4 human cell lines and 8 fresh human brain tumor biopsies. Currently, these biomarkers are assayed separately, and results can take days to weeks. We demonstrated the use of nanopore Cas9-targeted sequencing (nCATS) to identify *IDH1* and *IDH2* mutations within 36 h and compared this approach against currently used clinical methods. nCATS was also able to simultaneously provide high-resolution evaluation of *MGMT* methylation levels not only at the promoter region, as with currently used methods, but also at CpGs across the proximal promoter region, the entirety of exon 1, and a portion of intron 1. We compared the methylation levels of all CpGs to *MGMT* expression in all cell lines and tumors and observed a positive correlation between intron 1 methylation and *MGMT* expression. Finally, we identified single nucleotide variants in 3 target loci. This pilot study demonstrates the feasibility of using nCATS as a clinical tool for cancer precision medicine.

## Introduction

Diffuse gliomas (DG) comprise 80% of primary malignant central nervous system tumors in adults and traditionally were diagnosed with pathological criteria to define histological type (e.g., astrocytoma, oligodendroglioma, or oligoastrocytoma) and malignancy grade (e.g., grades I-IV) [1–3]. In 2016, the World Health Organization (WHO) diagnostic guidelines incorporated molecular markers into the classification of DGs [4, 5]. Many of these diagnostic biomarkers also serve as prognostic indicators, and the neuro-oncology community has supported this integration of molecular markers into clinical practice [6]. However, to date, there is wide variability in biomarker assessment because molecular techniques and test validity are inconsistent throughout the world and even within geographic regions [7, 8]. Therefore, the use of novel sequencing techniques that can assess multiple biomarkers simultaneously is an attractive option to overcome current clinical practice limitations. In this pilot study, we explore the use of nanopore Cas9-targeted sequencing (nCATS) to accomplish these goals.

To diagnose DG, the presence of isocitrate dehydrogenase 1 and 2 (*IDH1/2*) gene mutation is required for subtype identification and is also a prognostic molecular marker [4, 9]. The methylation status of the O6-methylguanine-DNA methyltransferase (*MGMT*) promoter is used routinely to guide chemotherapeutic treatment decisions, especially in glioblastoma (GBM) (e.g., grade IV astrocytoma), which is the most common type of DG. Thus, *IDH* and *MGMT* are the most commonly assayed molecular markers in patients with DG [10].

Various methods can be used to screen for *IDH1/2* mutation and *MGMT* promoter methylation. Typically, *IDH1/2* mutation screening is performed with an immunohistochemistry (IHC) assay specific for the most common mutation at* IDH*1 arginine 132 (arginine to histidine, R132H). However, IHC cannot detect other less common mutations, including *IDH*1 R132S, R132C, R132G, and R132L substitutions or IDH2 R172K. Polymerase chain reaction (PCR) or Sanger sequencing is thus recommended as a second-step test for IHC-negative tumors [4, 11].

Assaying *MGMT* methylation requires identifying the modification of cytosine residues on CpG islands (CpG methylation) in the promoter, which includes 98 CpG dinucleotides surrounding the transcription start site. These assays vary in the methodology used and the promoter region assessed. However, most interrogate only a fraction of the CpG sites to predict the transcriptional activity of the *MGMT* gene and in turn to predict potential therapeutic response to temozolomide (TMZ), an oral chemotherapy drug. Two differentially methylated regions (DMRs) cover CpGs 25–50 (DMR1) and CpGs 73–90 (DMR2) and have been demonstrated to correlate with transcriptional silencing [12]. DMR2 has some cis-acting sites that control the transcription of *MGMT* in a cell-based reporter study [13]. The presence of *MGMT* promoter methylation portends responsiveness to TMZ treatment [14, 15], but the degree of methylation corresponding to TMZ treatment response is a subject of debate, and there is no consensus on which assay method is optimal. Commonly used methods such as methylation-specific PCR, pyrosequencing, and mass spectrometry (MassARRAY®) introduce PCR bias and are restricted to study limited sequence length due to bisulfite treatment [16].

Nanopore technology (Oxford Nanopore Technologies® or ONT) could overcome the limitations of the aforementioned assays to assess both methylation and mutations. Quantitative methylation assessment without bisulfite conversion is possible with nanopore sequencing, as electrolytic current signals are sensitive to methylation of carbon 5 in cytosine (5mC) [17]. In addition, with the capacity for long-read single-molecule sequencing, multiple CpGs in the promoter region and additional surrounding regions can be captured. Here, we applied nanopore Cas9-targeted sequencing (nCATS) [18] and used the low-cost nanopore MinION device (ONT) to simultaneously assay *IDH* mutations and *MGMT* methylation. We also compared our results against currently used clinical tests. We observed a positive correlation between the methylation of all captured CpGs and gene expression levels and showed that both nCATS and existing deep sequencing methods detected the same single nucleotide variants in clinical DG samples.

## Materials and methods

### Informed consent

This study included 8 patients diagnosed with glioma. Case records were reviewed, and brain tissue samples were obtained under the approval of the institutional review board at the University of Arkansas for Medical Sciences (IRB protocol #228443). All patients provided written informed consent. Four samples with *IDH* mutations and 4 with *IDH* wild type were selected by A.R. However, all samples were processed and analyzed in a single-blind fashion before mutational status was disclosed to the analytical group (T.W. and P.J.).

### DNA samples and DNA extraction for nCATS

#### Control DNA

*IDH1/2* wild type gDNA standards (Horizon Discovery, USA) were used as the negative control for genotyping by PCR and nanopore sequencing (ONT, USA). For positive controls, *IDH1* codon 132 mutant DNA (CGT → GGT) was obtained from a patient in this study; *IDH2* codon 172 mutant DNA (AGG → AAG) was purchased from Horizon Discovery. Exon 4 of *IDH1/2* of each standard was amplified using specific primers (Integrated DNA Technologies, USA). PCR conditions for *IDH1/2* amplifications were identical, using 100 ng gDNA, 20 mM primers, and 25 μl LongAmp Taq 2x Master Mix (NEB, USA) with the following program: 95 °C 2 min, 25 cycles of [95 °C 15 s, 60 °C 30 s, 65 °C 40 s], 65 °C 10 min, 4 °C hold. PCR reactions were purified with AMPure XP beads (Beckman Coulter, USA) and eluted in 20 μl nuclease-free water (NEB). The purified PCR products were used for library preparation using 1D Native barcoding genomic DNA with EXP-NBD103 and SQK-LSK108 protocols (ONT) and nanopore sequencing with the R9.4.1/FLO-MIN106 flow cell (ONT).

The CpGenome™ DNA Standard Set (MilliporeSigma, USA) containing 5-mC and unmodified cytosines was used for quantitative analysis. The standard DNAs consist of linear, double-stranded DNA (897 bp) with 52 CpG sites; each standard contains either 100% 5-mCs or unmodified cytosines.

The CpGenome™ Human Methylated & Non-Methylated DNA Standard Set (MilliporeSigma) was used as the positive and negative control for nCATS and methylation status assessment. The Methylated DNA Standard is methylated enzymatically at all CpG dinucleotides (> 95%). The Non-Methylated DNA Standard contains less than 5% methylated DNA.

#### Cell line gDNA

Four GBM cell lines were used in this study: U87, U251, T98G, and LN18 (Sigma, USA). The cells were grown to 85–90% confluence in 10-cm dishes in DMEM (U87) with 10% fetal bovine serum (FBS); EMEM (U251 and T98G) with 2 mM glutamine, 1% NEAA, 1 mM sodium pyruvate, and 10% FBS; and in DMEM (LN18) with 5% FBS utilizing standard techniques. The cells were washed with PBS before DNA extraction with the AllPrep DNA/RNA Mini Kit (Qiagen, USA). Eluted gDNA was purified and concentrated using AMPure XP beads and eluted in 20–40 μl nuclease-free water and stored at − 20 °C.

#### Clinical samples

The study included 8 brain tissue samples graded according to the 2016 WHO classification for diffuse glioma by a board-certified neuropathologist, Murat Gokden M.D. (Table 1). Following surgical resection, tissue samples were immediately frozen on dry ice and stored at − 80 °C until DNA extraction. DNA extraction was carried out with the AllPrep DNA/RNA Mini Kit (Qiagen) as described above.

**Table 1:** Demographic characteristics of 8 patients

Patient ID	P553	P690	P701	P568	P785	P712	P816	P722
**Age**	29	24	57	42	72	37	48	73
**Gender**	Male	Male	Male	Male	Female	Female	Female	Female
**Race**	White	White	White	White	White	White	White	White
**Pathology Diagnosis**	Secondary GBM, WHO Grade 4	Secondary GBM, WHO Grade 4	Diffuse astrocytoma, WHO Grade 2	Diffuse astrocytoma, WHO Grade 2	Anaplastic astrocytoma, WHO Grade 3	Anaplastic astrocytoma, WHO Grade 3	GBM, WHO Grade 4	GBM, WHO Grade 4
**MGMT Status**	Low level detected	Detected	Detected	Detected	Not detected	Detected	Detected	Detected
**IDH**	Mutant	Mutant	Mutant	Mutant	Not detected	Not detected	Not detected	Not detected
**Previous Chemo**	Yes	Yes	No	No	No	No	No	No
**Chemo Agent**	TMZ	TMZ	NA	NA	NA	NA	NA	NA
**Previous Radiation**	Yes	Yes	No	No	No	No	No	No
**Previous Radiation Dose**	50.4Gy	60Gy	NA	NA	NA	NA	NA	NA
**Previous Diagnosis**	Diffuse astrocytoma, WHO Grade 2	Oligoastrocytoma, WHO Grade 2	NA	NA	NA	NA	NA	NA
**Progression Interval (months)**	30	55	NA	NA	NA	NA	NA	NA
**Vital Status**	Alive	Alive	Alive	Alive	Alive	Alive	Deceased	Deceased

### RNA extraction

For all cell lines and tissue samples, RNA and DNA were extracted from the same samples. The AllPrep DNA/RNA Mini Kit (Qiagen) allows the simultaneous purification of gDNA and total RNA from the same sample.

### Purity, quantity, and integrity of DNA and RNA

DNA and RNA purity was assessed in all samples with a NanoDrop-2000 spectrophotometer (Thermo Scientific, USA). DNA concentration was measured using a Qubit3.0 quantification assay (Thermo Scientific). The integrity of DNA and RNA was determined using a TapeStation 2200 (Agilent, USA).

### Single guide (sg)RNA design

To design the crRNAs, we used CHOPCHOP as described in the ONT protocol [19]. The specificity of the crRNA was tested with the UCSC In-Silico PCR tool to search against the human genome (hg19). The designed crRNAs, tracrRNA, and HiFi Cas9 were purchased from IDT. The following crRNAs were used: MGMT_promoter_left: ATGAGGGGCCCACTAATTGA; MGMT_promoter_right: ACCTGAGTATAGCTCCGTAC; IDH1_left: ACAGTCCATGAATCAACCTG; IDH1_right: GGCACCATACGAAATATTCT; IDH2_left: GCTAGGCGAGGAGCTCCAGT; IDH2_right: GCTGTTGGGGCCGCTCTCGA.

### nCATS library preparation for targeted sequencing by ONT

For each sample, 3.5 μg to 5.5 μg gDNA was used as input for preparing the nCATS library. The library preparation protocol was provided by ONT via the Enrichment Channel, Nanopore Community (protocol version: ENR_9084_v109_revA_04Dec2018). Briefly, gDNA ends were treated with calf intestinal phosphatase (NEB) to reduce the ligation of sequencing adapters to non-target strands. Then, Cas9 ribonucleoprotein complexes (Cas9 RNPs) were freshly prepared and used for generating double-strand breaks at targeted regions of blocked DNA. An adenine (A)-tail was immediately added to the 3′ ends of cut DNA fragments using Taq polymerase and dATP (NEB). The A overhang can pair with the T overhang of nanopore sequencing adapters. Both adapter-ligated DNA and blocked DNA were added to the flow cell for sequencing. The excess unligated adapters were removed with AMPure XP beads (Beckman Coulter). The library (molecules ligated to the adapters) were sequenced with the MinION Mk1B. Each library was sequenced for 36 h on an R9.4.1/FLO-MIN106 flow cell (ONT).

### Bioinformatics and statistical analysis

#### Data processing and mapping reads

The ONT raw signal data (FAST5 files) generated with MinKNOW software (version 1.7.14) were converted to DNA sequence data (FASTQ files) using the GUPPY algorithm (version 3.0.3). Quality control for ONT reads was performed to filter FASTQ files based on a mean quality threshold higher than Phred score 8 and read lengths longer than 200 bases using the NanoFilt program [20]. We aligned the filtered reads to the human reference genome (hg19) using Minimap2 and sorted them with SAMtools (version 1.6).

#### Nanopore methylation calling

CpG methylation (5mC) calling was performed with Nanopolish v 0.11.0 (17) using the reads (FASTQ files), aligned reads (BAM files), and raw signals (FAST5 files) for each sample. We then calculated the methylation frequency and log-likelihood ratios of methylation at each position using “calculate_methylation_frequency.py” from the Nanopolish package. We filtered out any position with < 10 reads and log-likelihood ratios of < 2.5 in each sample.

#### Single nucleotide variant calling

SNVs were called over the target regions with Nanopolish using FASTQ files, BAM files, and FAST5 files. Nanopolish was used to reanalyze the raw signals after alignment and to calculate SNV allele frequencies from the ONT data at the signal level. The “nanopolish variants” subprogram was used to simultaneously call SNVs with a modified parameter setting: -min-candidate-frequency = 0.15, −min-candidate-depth = 10,−-methylation-aware = cpg,−-snps, and --ploidy = 2. We reviewed the variant quality of SNVs and visualized them with the Integrative Genomics Viewer and trackViewer [21, 22].

### *MGMT* gene expression analysis with quantitative reverse transcriptase (qRT)-PCR

A total of 1 μg extracted RNA was reverse transcribed to cDNA using Superscript IV reverse transcriptase (Invitrogen, USA). qRT-PCR analysis was performed using iTaq Universal SYBR Green Supermix (BioRad, USA) and the StepOnePlus Real-Time PCR System (Applied Biosystems, USA). Real-time PCR was carried out in technical triplicates; it was run at 95 °C for 10 min, at 40 cycles of 95 °C for 15 s, and at 60 °C for 60s. A published primer set was used for *MGMT* and the β-actin gene (*ACTB*) [23–25]. For data analysis, the average result in each triplicate was used.

### Illumina sequencing of patient tumor samples

DNA and RNA sequencing was performed on clinical tumor specimens and saliva samples (from the same patients as the tumor specimens) for 6 of the 8 patients using the Tempus xT assay [26]. Briefly, nucleic acid was extracted from tumor tissue sections with tumor cellularity greater than 20% using a Chemagic360 instrument and a source-specific magnetic bead protocol. Total nucleic acid was used for DNA library construction, while RNA was further purified by DNase I digestion and magnetic bead purification. The nucleic acid was quantified with a Quant-iT PicoGreen dsDNA Kit or Quant-iT RiboGreen RNA Kit (Life Technologies), and quality was confirmed with a LabChip GX Touch HT Genomic DNA Reagent Kit or LabChip RNA High HT Pico Sensitivity Reagent Kit (PerkinElmer).

For DNA library construction, 100 ng DNA from tumor or normal samples was mechanically sheared to an average size of 200 bp using a Covaris ultrasonicator. The libraries were prepared using the KAPA Hyper Prep Kit. Briefly, DNA underwent enzymatic end repair and A-tailing, followed by adapter ligation, bead-based size selection, and PCR. The captured DNA targets were amplified using the KAPA HiFi HotStart ReadyMix. The amplified target-captured libraries were sequenced on an Illumina HiSeq 4000 System with patterned flow cell technology.

## Results

### Nanopore sequencing accurately assesses mutational status and methylation levels

**Fig. 1:**
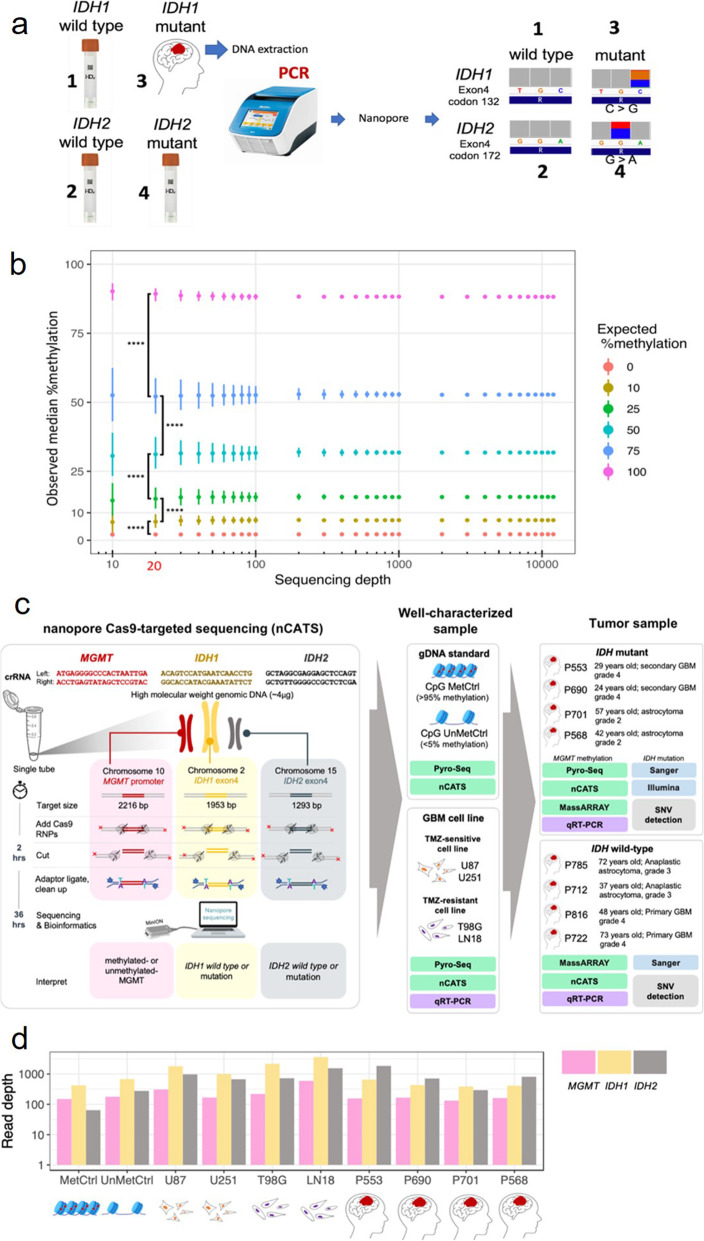
Mutation and methylation assessments with well-characterized samples was used to develop nCATS workflow. **a**, Genotyping of *IDH1* wild type (purchased), *IDH2* wild type (purchased), *IDH2* R172K mutation (purchased), and *IDH1* R132G mutation (fresh biopsy sample). Exon 4 of *IDH1* and *IDH2* were PCR amplified and sequenced with nanopore technology. Nanopolish correctly genotyped all samples. **b,** Observed and expected CpG methylation percentage detected on methylated and unmethylated DNA standards. Standards that were 100% methylated or 0% methylated on CpGs were sequenced, and methylation calling was performed with Nanopolish. Data were generated for 10, 25, 50%, or 75% methylated CpGs by randomly sampling reads from each standard; at ≥20 depth coverage (20X), methylation levels of 0, 25, 50, 75, and 100% could be distinguished. Data represent the median, with 25th and 75th percentiles. Pairwise *t*-test with Bonferroni correction **** *P* < 0.0001. Thus, 20X was used as the theoretical limit of detection in this study. **c,** Guide RNA (crRNA) for 3 target loci (*MGMT*, *IDH1*, and *IDH2*) were designed and used for nanopore Cas9-targeted sequencing (nCATS) with the MinION device. Various types of sample were used for testing the feasibility of nCATS to assay methylation and mutations. GBM, glioblastoma; TMZ, temozolomide. **d,** Median coverage of each loci for 10 samples

The error rate of raw nanopore sequencing reads continues to decrease, allowing the technology to be used for genotyping and methylation assays [17]. Nanopore sequencing errors are largely random and use of a consensus sequence from sufficient read depth can eliminate almost all of the sequencing error. To confirm the ability of nanopore sequencing to accurately genotype the *IDH* mutations, we sequenced PCR amplicons that were *IDH1/2* wild type or *IDH1/2* mutant using a nanopore MinION device. This test showed that heterozygous mutations in these 2 genes could be accurately detected, although artificial errors are inevitable (Fig. 1a).

To determine the limit of detection for CpG methylation, we sequenced 2 synthetic DNA standards with that were either 100% methylated or 0% methylated on CpGs and then used Nanopolish for methylation calling [17]. We generated data for 10, 25, 50%, or 75% methylated CpGs by randomly sampling the reads from the 0 and 100% methylated standards. We found that at a low sequencing coverage of ~ 10 reads (10X), methylation could be measured, but with high variation. Decreasing of coefficient of variation when increasing of sequencing depth was observed (Supplementary Table 1). At higher depth, ≥20X, the standard deviation was lower (Fig. 1b), and methylation levels of 0, 25, 50, 75, and 100% could be distinguished. Thus, 20X was used as the theoretical limit of detection in this study.

### nCATS *MGMT* methylation assay is comparable to pyrosequencing assays

Based on these preliminary data, we then designed guide RNA for the nanopore Cas9-targeted sequencing (nCATS) workflow (18) to test on 4 human GBM cell lines (2 TMZ-sensitive [U87 and U251]) and 2 TMZ-resistant [T98G and LN18] and 8 clinical DG samples (4 *IDH* mutant and 4 *IDH* wild type) (Fig. 1c and Table 1). Sequencing depth coverage was an average of 184, 664, and 939 for *MGMT*, *IDH1*, and *IDH2*, respectively (Fig. 1d).

**Fig. 2:**
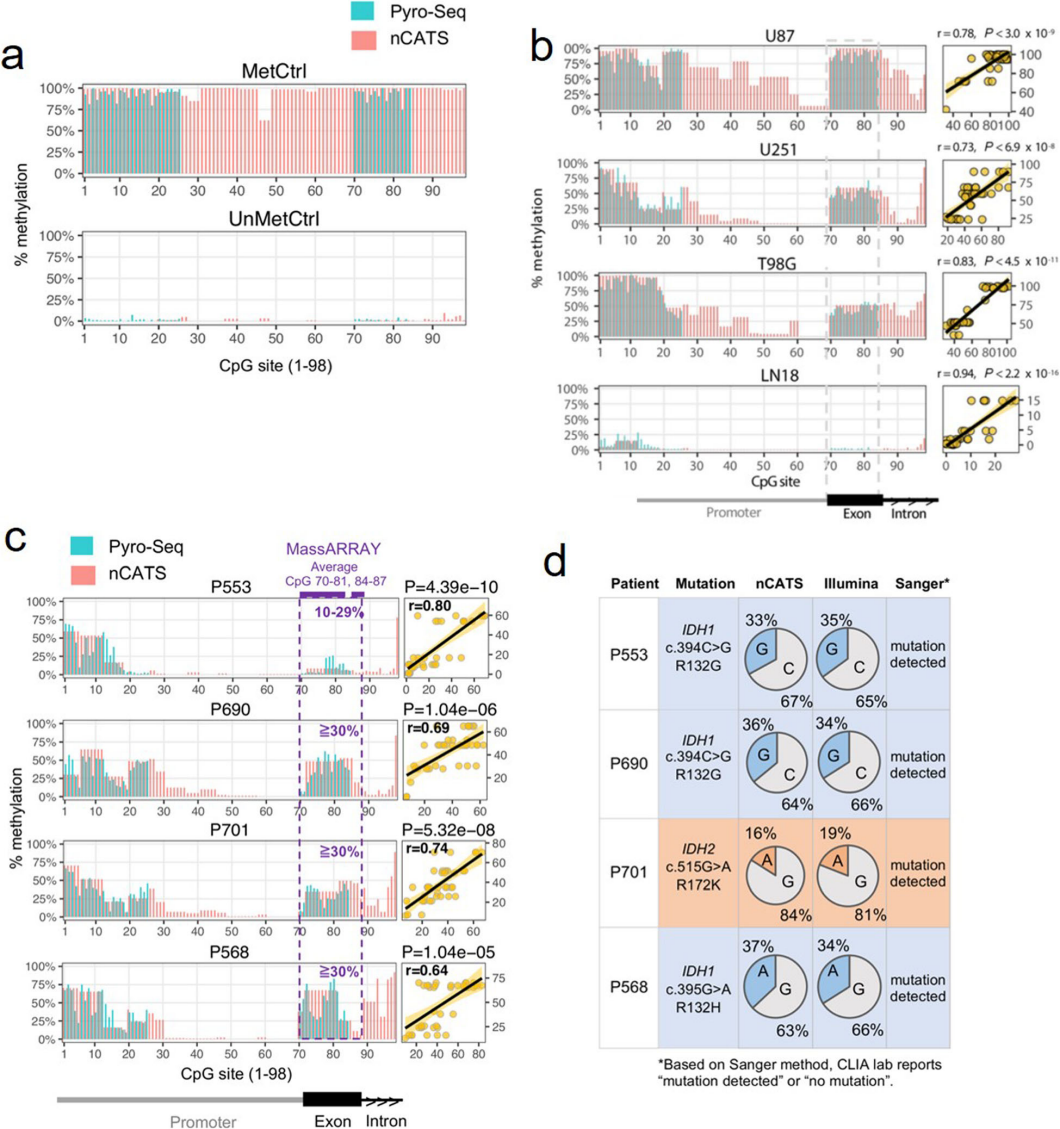
Simultaneous assessment of *MGMT* and *IDH* status in 4 *IDH*-mutant clinical samples. **a,** Methylation was assayed by pyrosequencing and nCATS in 2 DNA standards: CpG methylated (MetCtrl) and unmethylated (UnMetCtrl). **b,** Methylation was assayed in DNA extracted from 4 glioblastoma cell lines: U87, U251, T98G, and LN18. Correlation (r) of methylation level between nCATS and pyrosequencing was calculated with *P*-value. Each yellow point is an individual CpG. **c,** Methylation pattern was assayed by pyrosequencing, MassARRAY, and nCATS in 4 *IDH*-mutant clinical samples. Correlation (r) of methylation level between nCATS and pyrosequencing was calculated with *P*-value. Each yellow point is an individual CpG. **d,***IDH* mutations were detected with the nCATS, Illumina, and Sanger sequencing platforms. *IDH1* mutations were accurately detected in 3 patients (blue rows), and *IDH2* mutation was detected in 1 patient (orange row). The pie charts and percentages indicate allele frequency detected by each method

We then used nCATS to perform targeted sequencing of the *MGMT* gene; this approach captured 98 CpGs (located in promoter and exon 1) and 121 CpGs (in a 5’end of intron 1). The genomic coordinates of CpG loci are shown in Supplementary Table 2. The first 98 CpGs have been studied by others, and a subset of CpGs in this region has been used clinically to assess methylation [16]. Thus, we first focused on the 98 CpGs and used them to compare the methylation levels obtained by nCATS to levels obtained by pyrosequencing assays. Using a methylated and unmethylated DNA standard with > 95% vs < 5% methylation, respectively, nCATS provided a clear methylation pattern in both samples (Fig. 2a) that was comparable to the results of bisulfite modification-PCR-pyrosequencing for CpGs 1–25 and 70–84 (detail in Supplementary Tables 2 and 3).

We next applied nCATS to 4 well-characterized GBM cell lines (described above). The percent methylation of these 4 cell lines assayed by nCATS also correlated positively (r = 0.73, *P* = 6.9 × 10^− 8^ to r = 0.94, *P* = 2.2× 10^− 16^) with the percent methylation returned by pyrosequencing (Fig. 2b). At this point, we concluded that methylation data derived from nCATS is comparable to data derived from pyrosequencing assays when applied to a homogeneous sample (e.g. an immortalized glioma cell line).

### Simultaneous evaluation of methylation and mutation biomarkers in patients with diffuse glioma

We next confirmed that nCATS can be used in clinical samples that have heterogenous cell populations opposed to the glioma cell lines. To test the accuracy of nCATS to assay *MGMT* methylation and *IDH1/2* mutations in clinical samples. For *MGMT* methylation, we compared the nCATS data to data generated with bisulfite modification-PCR-pyrosequencing or the MassARRAY® System performed by 2 independent Clinical Laboratory Improvement Amendments (CLIA)-certified labs. There was a statistically significant positive correlation (r = 0 0.64, *P* = 1.04 × 10^− 5^ to r = 0.80, *P* = 4.39 × 10^− 10^) between nCATS quantitative methylation and pyrosequencing (Fig. 2c). MassARRAY® results were semiquantitative and only denoted methylation levels in 3 categories (not detected: < 10%; low methylation: 10–30%; detected: > 30%) for CpG sites 70–81 and 84–87. These MassARRAY® results also showed a similar trend with nCATS results over the same CpG sites.

The sample from patient 553 had 8% methylation over the targeted CpG sites, and MassARRAY® determined it to have a low level of methylation. In the other 3 patients, methylation ranged from 38 to 51%, and MassARRAY® reported “detected” methylation (i.e., > 30%) (Fig. 2c). It is worth noting that fresh biopsies were used for nCATS and pyrosequencing, while formalin-fixed, paraffin-embedded samples were used in the MassARRAY® System.

With respect to detecting *IDH* mutations, nCATS showed *IDH* mutations in all patient samples consistent with Sanger (CLIA-certified lab) and exome sequencing (Illumina) data. The allele frequencies detected by nCATS and Illumina were similar (within ±3%), *P* = 0.91892 (chi-squared test) (Fig. 2d).

### *MGMT* expression negatively correlates with *MGMT* exon methylation but positively correlates with *MGMT* intron methylation

**Fig. 3:**
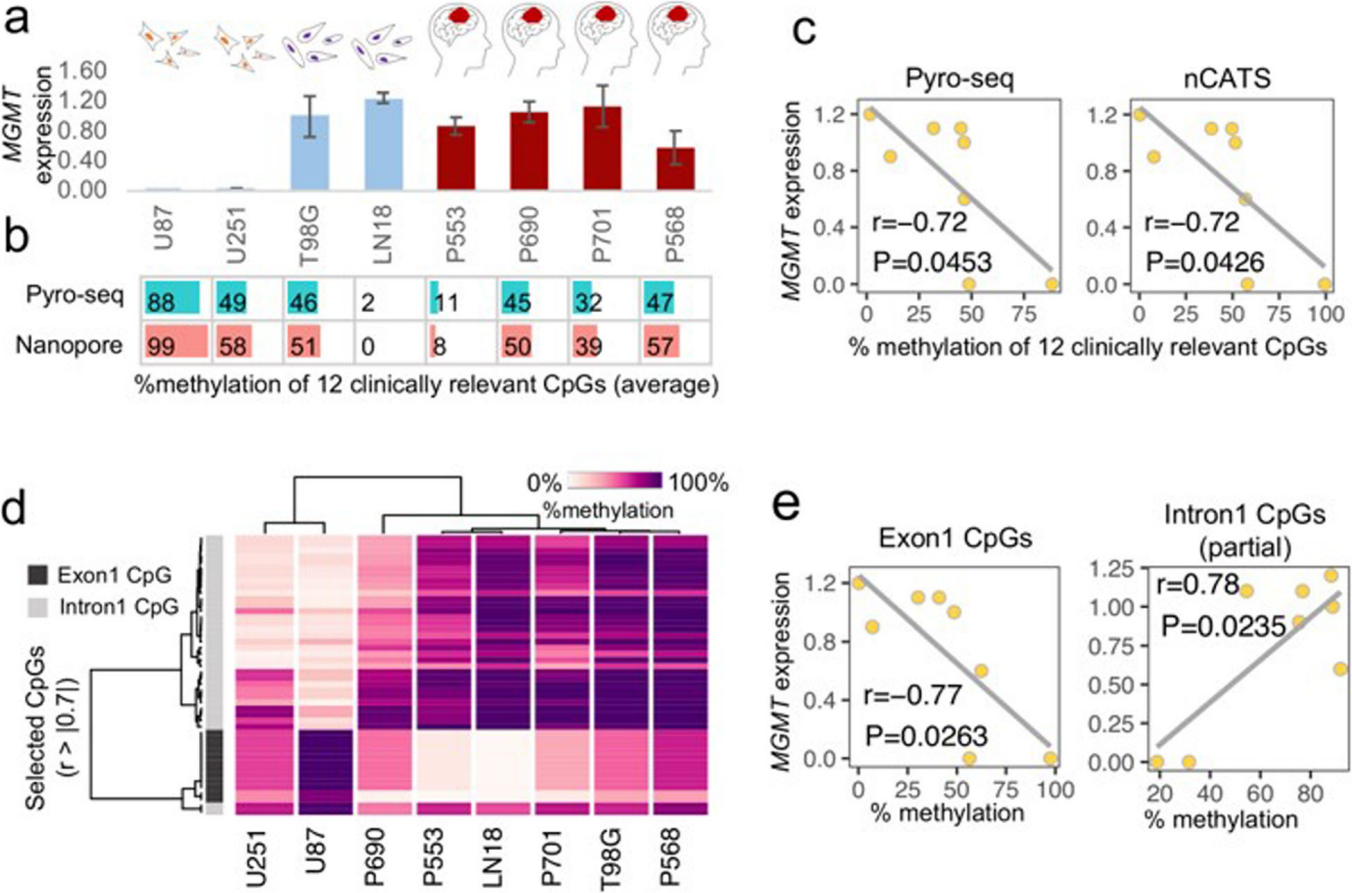
Correlation between *MGMT* gene expression and CpG methylation at different loci. **a,***MGMT* gene expression was measured with qRT-PCR in 4 cell lines and 4 *IDH*-mutant tumor samples. Data are the mean ± SD (3 technical replicates). **b,** Percent methylation of 12 clinically relevant CpG sites within *MGMT* exon 1. **c,** Correlation between *MGMT* expression and methylation detected by pyrosequencing vs. nCATS. Each yellow point is an individual sample. **d,** Heat map and hierarchical clustering of percent methylation of the exon 1 CpGs and a portion of the intron 1 CpGs. Selected CpGs (r > 0.7 or r < − 0.7) were used for clustering. **e**, Correlation between *MGMT* expression and exon 1 methylation and between *MGMT* expression and intron 1 methylation

We next determined the relationship between *MGMT* gene expression and *MGMT* methylation level in the 4 cell lines and 4 tumor samples. *MGMT* expression negatively correlates to TMZ clinical response. A total of 12 CpGs in differentially methylated region 2 (DMR2, in this study CpGs 70–81 in exon 1) were considered because not only could we compare nCATS and pyrosequencing data, but these CpGs are clinically relevant. As expected, qRT-PCR demonstrated high *MGMT* expression in TMZ-resistant cell lines and very low *MGMT* expression in TMZ-sensitive cell lines (Fig. 3a). An inverse correlation between *MGMT* expression and methylation (Fig. 3b) was shown with both nCATS and pyrosequencing (r = − 0.72), with similar significance levels (*P* < 0.05) (Fig. 3c). These data suggested that in general nCATS produced sequencing data comparable to that of conventional methods.

We further investigated each sample in detail and found an unexpected result in the T98G cell line. Although, we observed high expression of *MGMT* as previous studies [27] but observed methylation level and gene expression were not opposed (Fig. 3a and b). This unexpected result led us to investigate the methylation of additional CpGs with nCATS (CpG 99–219). CpGs that had strong correlation (r > 0.7 or r < − 0.7) between *MGMT* expression and methylation were selected for by clustering analysis including 12 CpGs in the exon 1 and 34 CpGs in the intron 1. Hierarchical clustering according to CpG sites showed 2 clear position-dependent clusters: CpGs in exon 1 were clustered together and separated from CpGs in intron 1 (Fig. 3d). Hierarchical clustering of the 8 samples (4 cell lines and 4 tumors) demonstrated 2 distinct clusters: 2 TMZ-sensitive cell lines with similar methylation profiles were clustered together, while 2 TMZ-resistant cell lines and the 4 clinical samples were clustered together (Fig. 3d). Moreover, we found that intronic CpG methylation positively correlated with *MGMT* expression (r = 0.78, *P* = 0.024); whereas, exonic CpG methylation remained negatively correlated with *MGMT* expression (r = − 0.77, *P* = 0.026) (Fig. 3e).

**Fig. 4:**
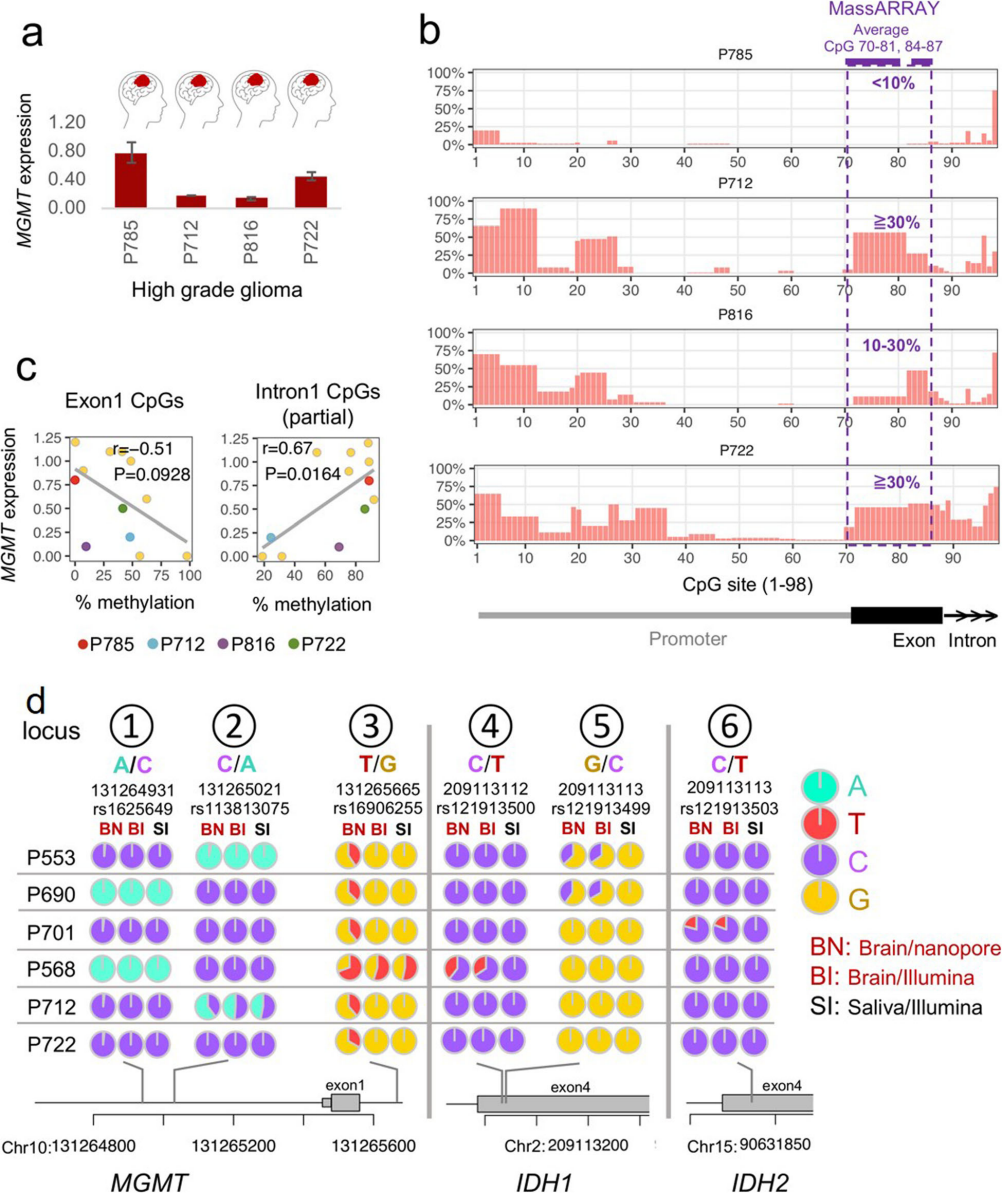
nCATS can simultaneously quantify *MGMT* CpG methylation and detect Single nucleotide variants (SNVs) in glioma clinical samples. **a,***MGMT* gene expression in 4 *IDH* wild type samples by qRT-PCR. Data are the mean ± SD (3 technical replicates). **b,** Methylation pattern by nCATS and MassARRAY. **c,** Correlation between *MGMT* expression and exon 1 methylation and between *MGMT* expression and intron 1 methylation. **d,** SNVs in *MGMT* and *IDH1/2* were assayed with nCATS and Illumina sequencing in tumor and saliva samples from 6 patients. Data were plotted with trackViewer. No data were available for P785 and P816

To test additional tumor grades, 4 tumor samples classified as primary WHO grade III or IV (high-grade gliomas) were assayed with qRT-PCR for *MGMT* expression and nCATS for methylation. These 4 samples differed from the previous clinical samples not only in tumor classification, but they came from *IDH* wild type patients. *MGMT* expression (Fig. 4a) and *MGMT* methylation pattern (Fig. 4b) varied between samples. The data for these 4 samples were combined with data for the 8 previous samples (including cell lines) for correlation analysis. With 12 samples, a negative correlation between *MGMT* expression and methylation in exon 1 was present (r = − 0.51) but not statistically significant (*P* = 0.093). However, there was a statistically significant positive correlation for *MGMT* expression and methylation in intron 1 (r = 0.67, *P* = 0.016) (Fig. 4c). For *IDH* genotyping in these last four clinical samples, nCATS detected *IDH1* and *IDH2* as wild type, consistent with Illumina and Sanger sequencing results.

### nCATS identified single nucleotide variants

Finally, we showed that nCATS could be used to identify single nucleotide variants (SNVs) in *MGMT* and *IDH1/2* loci (Fig. 4d). We compared nanopore sequencing with Illumina sequencing and also verified the absence of the pathogenic SNVs in germ-cell DNA using Illumina-sequenced saliva samples from 6 of the patients (no Illumina data available for P785 and P816). nCATS and Illumina returned similar genotypes for *MGMT* loci 1 and 2 (Fig. 4d). For locus 2, both methods detected heterozygous alleles (C/A) in both tumor and saliva from Patient 712. For locus 3, nCATS detected heterozygous alleles in all samples, while Illumina showed heterozygous alleles in only 1 sample. For loci 4, 5 (*IDH1*), and 6 (*IDH2*), nCATS and Illumina consistently detected somatic variants (the variants were not identified in saliva samples).

## Discussion

In this study, we used nanopore Cas9-targeted long-read sequencing (nCATS) to simultaneously assess 2 prognostic molecular markers in diffuse glioma clinical samples and cell lines—*MGMT* methylation and *IDH1/2* mutations. nCATS enables enrichment of genomic regions without amplification [18, 28], quantitative analysis of methylation on native DNA, and identification of single nucleotide variants. Gilpatrick et al. assessed clinical cancer biomarkers (e.g., *TP53*, *KRAS*, and *BRAF*) with nCATS in breast cancer cell lines and 1 patient tumor sample, demonstrating its feasibility [18]. Here, we demonstrated the feasibility of using nCATS on several clinical solid tumor samples to assess both genetic and epigenetic prognostic biomarkers that are clinically relevant.

nCATS allowed for simultaneous evaluation of *IDH1/2* mutational status and *MGMT* methylation level in a streamlined workflow, resulting in biomarker assessment within 36 h (Fig. 1c). The ability of nanopore sequencing to evaluate methylation from native DNA sequences obviated the need for bisulfite modification [17], and we were able to achieve adequate depth coverage without amplification even in clinical samples. Our assessment of *IDH* mutational status correlated with clinically used Sanger methods and was further compared with Illumina sequencing (Fig. 4d).

*MGMT* methylation assessment is currently highly variable, as both the methodology used and the gene region evaluated are not consistent between clinicians. Further, no cutoff value in *MGMT* methylation level has been verified to correlate with *MGMT* expression; thus, no clinical consensus exists [16, 29]. Many institutions evaluate 2 differentially methylated regions (DMRs) within the *MGMT* promoter and exon 1 that have been shown to correlate with *MGMT* expression in cell lines and patient cohorts [13, 30]; *MGMT* methylation is then used to predict responsiveness to temozolomide (TMZ) therapy. Our institution uses MassARRAY® and stratifies patients into 3 groups: no methylation (< 10%), low methylation (10–30%), and high methylation (> 30%). In this study, nCATS data from both cell lines and patient samples correlated with both MassARRAY® data and pyrosequencing (Fig. 2c and 4b). However, some patients who are below this arbitrary cutoff value (e.g., 10%) do respond to TMZ therapy [31–33], placing them in a “gray zone” and producing a clinical quandary. With this in mind, Chai et al. developed a novel CpG averaging model for pyrosequencing data that defines the *MGMT* promoter as being methylated when at least 3 CpGs exceed their respective cutoff values; this allows clinicians to better stratify patients with very low levels of methylation (e.g., < 10%) [34]. We demonstrate that nCATS can be used to quantify CpG methylation in multiple regions of the *MGMT* gene and may provide further insight into the variability of treatment responses. In the future, this long-read sequencing method could provide a reliable and thorough quantitative assessment of *MGMT* to develop a cutoff methylation value, but a large validation cohort will be needed.

Given the long-read sequencing capacity of nCATS, we were also able to quantify CpG methylation along the entire *MGMT* promoter, exon 1, and a portion of intron 1. One of the TMZ-resistant cell lines (T98G) did not have the expected inverse correlation between *MGMT* promoter methylation level and *MGMT* expression. There was a positive correlation between methylation of intronic CpG sites and *MGMT* expression for all GBM cell lines, the *IDH* mutant sample, and wild type DG samples (Fig. 3e and 4c). The result was in agreement with recent studies demonstrating the role of CpG methylation in the gene body (outside the promoter) in regulating levels of *MGMT* gene expression, with higher levels of gene body cytosine modification correlating with higher *MGMT* expression [27, 35]. This finding suggests a potential benefit of assaying gene body methylation, as the intron could be important for determining *MGMT* expression; however, a larger sample size is needed.

Finally, we identified 2 SNVs in the promoter region of *MGMT*, and one of them (rs1625649) had prognostic impact on patients with MGMT methylated glioblastoma [36, 37]. In *MGMT*, inconsistency between nCATS and Illumina result was also observed. In locus no.3 (Fig. 4d), nCATS detected 2 alleles in all patients while Illumina showed 2 alleles in only P568. We then considered the DNA sequence in this region and found 6 consecutive guanines (homopolymer) in this locus. For the current version of nanopore, homopolymer rich regions are the major source of errors. Therefore, for this locus, nCATS could not deliver accurate genotyping when using this version of nanopore (R9.4.1). An updated version of nanopore is being developed that incorporates a longer sensor to overcome errors in homopolymer rich regions.

Our nCATS technique also identified mutation variants (locus no.4–5 (Fig. 4))in *IDH1* and *IDH2*. The variants in *IDH1* are associated with survival in patients with acute myeloid leukemia [38], but their prognostic value in GBM is not known [39]. However, with the advent of new *IDH*-directed therapies, variants in *IDH1/2* may be of significance in the future [40]. These insights could lead to the incorporation of SNVs as an additional factor in therapeutic decision making, which can be done contemporaneously along with biomarker identification with nCATS.

In conclusion, the nCATS technique provides results within 2 days of surgical resection, potentially at lower capital cost than traditional methods. We demonstrated feasibility in clinical solid tumor samples and used DG as a model given that both genetic and epigenetic biomarkers are used clinically. The nCATS method also provided assessment of *MGMT* methylation throughout a larger gene region in comparison to currently used methods. There is great potential to use nCATS clinically to standardize molecular marker testing in DG and provide insights into patient variability to treatment response. Furthermore, nanopore platforms can be cost-effective and high-throughput, making them accessible in countries with limited resources [41]. In the future, we plan to design a comprehensive nCATS based DG diagnostic panel and testing on larger prospective cohorts. nCATs requires > 3 μg of high-quality DNA as starting material, making testing formalin-fixed specimens impractical. Obtaining tissue from fresh samples requires consideration of choosing a region with low necrosis and high tumor content in order to optimize DNA extraction. Nevertheless, the nCATS method provides a promising tool for enhancing cancer precision medicine with the potential for simultaneously assessing multiple molecular targets.

## Supplementary information

**Additional file 1.**

## Data Availability

All raw signal data (FAST5 files) for this study have been deposited in the European Nucleotide Archive under the study accession PRJEB33258. The base-called data (FASTQ files) have been deposited in the Genbank database under BioProject ID: PRJNA549038
